# Supplementary material for: Numerical and analytical results for geometric measure of coherence and geometric measure of entanglement
Source: Sci Rep. 2020 Jul 21;10:12122. doi: 10.1038/s41598-020-68979-z (PMC7374728; doi:10.1038/s41598-020-68979-z)
Supplement: Supplementary file 1 [file 41598_2020_68979_MOESM1_ESM.pdf]

# Supplemental Material: Numerical and analytical results for geometric measure of coherence and geometric measure of entanglement

Zhou Zhang, Yue Dai, and Yuli Dong*
*School of Physical Science and Technology, Soochow University, Suzhou, 215006, China*

Chengjie Zhang†
*School of Physical Science and Technology, Ningbo University, Ningbo, 315211, China and*
*School of Physical Science and Technology, Soochow University, Suzhou, 215006, China*

Here we provide some detailed calculations of the single-qubit states, randomly generate 3-dimensional density matrices, and a special kind of $d$-dimensional density matrices for coherence measures. The corresponding MATLAB code for the semidefinite program of geometric measure of coherence is

```
function [C, D]=coherence(rho)

   d=length(rho);

   delt=sdpvar(d,d, 'diagonal', 'real');

   X=sdpvar(d,d, 'full', 'complex');

   constr=[[rho X; ctranspose(X) delt]≥0, delt≥0, trace(delt)==1];

   result=solvesdp(constr, -trace(X)-trace(ctranspose(X)), sdpsettings('verbose', 1));

%check for errors
   if (result. problem ≠ 0 )
      disp(result. info);
   end

%return the maximum fidelity
   F=double((trace(X)+trace(ctranspose(X)))/2);

   D=double(delt);

   C=1-F^2;

end
```

We have used the parser YALMIP [1] with the solvers, SEDUMI [2] or SDPT3 [3].

We also provide some detailed calculations of the two-qubit states, the isotropic states and the Werner states for entanglement measures. The corresponding MATLAB code for the semidefinite program of geometric measure of entanglement is

```
function [E, E2]=GME(rho)

%d(i) is the dimension of the matrix
   d1=2;
   d2=2;

   sigma=sdpvar(d1*d2, d1*d2, 'hermitian', 'complex');

   X=sdpvar(d1*d2, d1*d2, 'full', 'complex');

%performing partial transpose for sigma and the pt subroutine comes from pptmixer.
   constr=[[rho X; ctranspose(X) sigma]≥0, sigma≥0, pt(sigma, [1,0], [d1, d2])≥0, trace(sigma)==1];
```

*Electronic address: yldong@suda.edu.cn
†Electronic address: chengjie.zhang@gmail.com

```

   result=solvesdp(constr, -trace(X)-trace(ctranspose(X)), sdpsettings('verbose', 1));

%check for errors
   if (result. problem ≠ 0)
       disp(result. info);
   end

%return the maximum fidelity
   F=double((trace(X)+trace(ctranspose(X)))/2);

   D=double(sigma);

   E=1-F^2;

end
```

We have used the pt subroutine from the program PPTmixer, where the PPTmixer was presented in [4] (or [5]).


[1] https://yalmip.github.io
[2] http://sedumi.ie.lehigh.edu
[3] http://www.math.nus.edu.sg/~mattohkc/sdpt3.html
[4] B. Jungnitsch, T. Moroder and O. Gühne, Phys. Rev. Lett. **106**, 190502 (2011).
[5] See the program PPTmixer, (available at www.mathworks.com/matlabcentral/fileexchange/30968).
